# Diminishing Returns from Increased Percent Bt Cotton: The Case of Pink Bollworm

**DOI:** 10.1371/journal.pone.0068573

**Published:** 2013-07-16

**Authors:** Yunxin Huang, Peng Wan, Huannan Zhang, Minsong Huang, Zhaohua Li, Fred Gould

**Affiliations:** 1 School of Resource and Environmental Science, Hubei University, Wuhan, P. R. China; 2 Institute of Plant Protection and Soil Science, Hubei Academy of Agricultural Sciences, Wuhan, P. R. China; 3 Department of Entomology, North Carolina State University, Raleigh, North Carolina, United States of America; French National Institute for Agricultural Research (INRA), France

## Abstract

Regional suppression of pests by transgenic crops producing insecticidal proteins from *Bacillus thuringiensis* (Bt) has been reported in several cropping systems, but little is known about the functional relationship between the ultimate pest population density and the pervasiveness of Bt crops. Here we address this issue by analyzing 16 years of field data on pink bollworm (*Pectinophora gossypiella*) population density and percentage of Bt cotton in the Yangtze River Valley of China. In this region, the percentage of cotton hectares planted with Bt cotton increased from 9% in 2000 to 94% in 2009 and 2010. We find that as the percent Bt cotton increased over the years, the cross-year growth rate of pink bollworm from the last generation of one year to the first generation of the next year decreased. However, as the percent Bt cotton increased, the within-year growth rate of pink bollworm from the first to last generation of the same year increased, with a slope approximately opposite to that of the cross-year rates. As a result, we did not find a statistically significant decline in the annual growth rate of pink bollworm as the percent Bt cotton increased over time. Consistent with the data, our modeling analyses predict that the regional average density of pink bollworm declines as the percent Bt cotton increases, but the higher the percent Bt cotton, the slower the decline in pest density. Specifically, we find that 95% Bt cotton is predicted to cause only 3% more reduction in larval density than 80% Bt cotton. The results here suggest that density dependence can act against the decline in pest density and diminish the net effects of Bt cotton on suppression of pink bollworm in the study region. The findings call for more studies of the interactions between pest density-dependence and Bt crops.

## Introduction

Use of transgenic crops that produce insecticidal proteins derived from *Bacillus thuringiensis* (Bt) has become a major strategy for controlling Lepidoptera pests since 1996. In 2011, more than 66 million hectares of Bt cotton and Bt corn were planted worldwide [1], [2]. Widespread adoption of Bt crops has brought up several benefits such as reduced crop damage, reduced insecticide sprays and enhanced biocontrol services [3]. One serious threat to the sustainable use of Bt crops, however, is the evolution of resistance by pests [4]–[7]. The main strategy for delaying pest resistance to Bt crops is planting of non-Bt host plants near Bt crop fields as refuges to promote survival of susceptible pests [5], [8], [9].

One of the potential issues with the refuge strategy is pest damage to non-Bt plants. However, a few documented cases suggest that Bt plants can help suppress pest on nearby non-Bt plants, as a result of a “halo effect” [10]–[18]. The rational is that females emerging from non-Bt plants lay some of their eggs on nearby Bt plants, and the larvae hatching from such eggs suffer high mortality on the Bt plants [11], [13]. If Bt plants account for a substantial percentage of the available host plants, regional pest populations can be greatly reduced, resulting in less damage to non-Bt plants [11].

The halo effect has been documented for pink bollworm (*Pectinophora gossypiella*) in the United States [13], a widespread pest that feeds primarily on cotton [19]–[21]. Analysis of 10 consecutive years of field data beginning five years before the adoption of transgenic cotton varieties that produce the Bt toxin, Cry1Ac, in the state of Arizona (USA) shows that regional suppression of pink bollworm in non-Bt cotton is achieved when the percentage of cotton planted with Bt cotton exceeds a threshold of approximately 65% [13]. The halo effect has also been reported for the polyphagous cotton bollworm (*Helicoverpa armigera*) in Northern China. Analysis of 10 years of field data show that as the planting area of Bt cotton increases annually, cotton bollworm densities have gradually declined, not only in cotton plantings but also on other non-Bt host plants [16]. Another example of the halo effect involves the European corn borer (*Ostrinia nubilalis*) in Minnesota, Illinois and Wisconsin (USA). Data analyses show that the annual pest densities on non-Bt maize have declined since Bt maize was commercialized [17].

The halo effect was recently reexamined for pink bollworm in the six provinces of the Yangtze River Valley of China [22]. In this region, Bt cotton was not introduced until 2000. The percentage of cotton hectares planted with Bt cotton increased from 9% in 2000, to 62% in 2005, 84% in 2006, and 94% in 2009 and 2010. Analyses of field data have shown that as the percent Bt cotton increases over time, the annual average population densities of pink bollworm on non-Bt cotton plants decrease significantly [22].

While the halo effect for pink bollworm has demonstrated that pest suppression on non-Bt cotton can be achieved with intensive planting of Bt cotton, the functional relationship between the percent Bt cotton and pest density over a long time duration has never been established. It is clear that increasing the percent Bt cotton causes a trade-off between the short-term benefit of reduced pest damages and the long-term risk of pest resistance to Bt cotton. If there is some point at which increasing the percent Bt cotton does not cause a further substantial decrease in pest population density or pest damage, then the risk of pest resistance can be reduced by minimizing the percent Bt cotton to that point.

**Figure 1:**
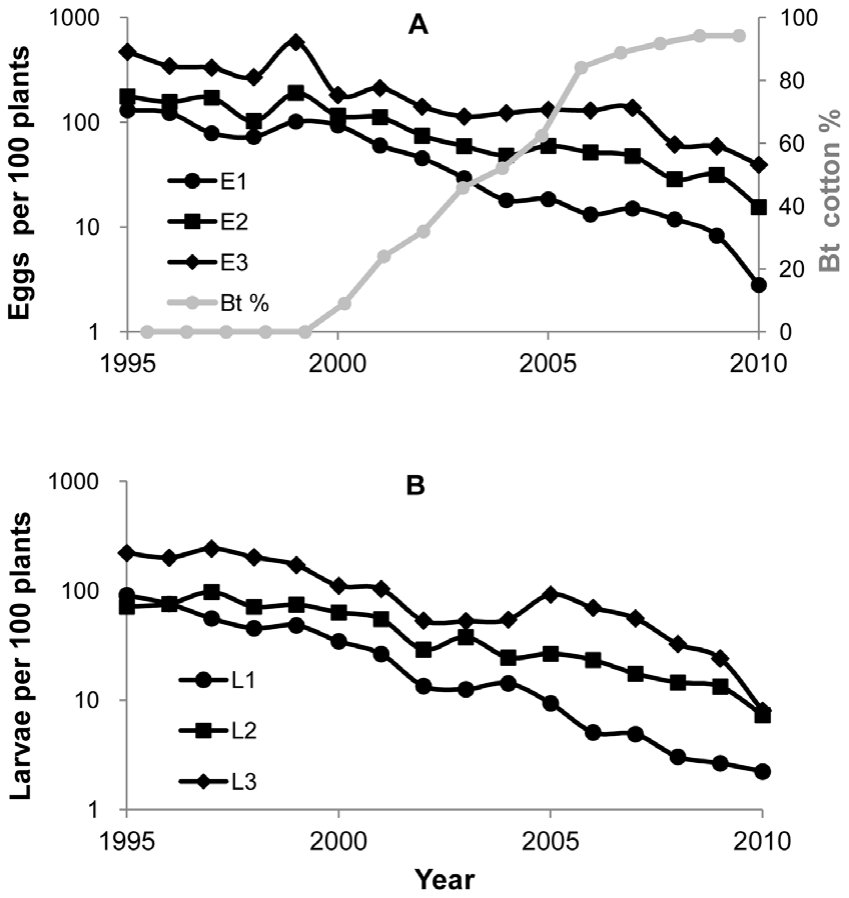
Population abundance of pink bollworm on non-Bt cotton and the percent Bt cotton planting area in the Yangtze River Valley. (**A**) Average number of pink bollworm eggs per 100 plants in the first, second and third generation (indicated by E1, E2 and E3 respectively and plotted on y-axis to the left) and percent Bt cotton (plotted on y-axis to the right). (**B**) Average number of pink bollworm larvae per 100 plants in the first, second and third generation (indicated by L1, L2 and L3 respectively).

To address these issues we examine the detailed data on density of pink bollworm (*Pectinophora gossypiella*) in each generation for six provinces of the Yangtze River Valley of China from 1995 to 2010 (Fig. 1). The same data have been reported previously [22], with the goal of addressing whether the annual average population density of pink bollworm had declined over time. In that work, within season dynamics were not assessed. Here we report these details and use them to analyze the within season and overwintering dynamics of pink bollworm. These in-season and between-season dynamics are essential for understanding the functional relationship between the percent Bt cotton and pest density. We show that direct density dependence may act against the decline in pest density, diminishing the net effects of Bt cotton on suppression of pink bollworm.

## Results

### Annual Pink Bollworm Growth Rates

The percentage of cotton hectares planted with Bt cotton in the Yangtze River Valley increased from 9% in 2000 to 94% in 2009 and 2010, with a rate of increase approximately 10% per year (Fig. 1; see also [22]). For eggs and larvae, there was a difference in annual per-capita growth rate (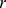) when contrasting all years in which the percent Bt cotton was more and less than 65% [22], but the regression of annual growth rate against percent Bt was not significant (*P*>0.15; see Table 1). This result suggests that the annual population density of pink bollworm declined in the later years when the percent Bt cotton was high, but there might exist an inherently diminishing return as the percent Bt cotton increased over time. To examine this more closely we decomposed the annual growth rate into a within-year growth rate and a cross-year growth rate (see Methods) and examined them separately.

**Table 1:** Test results for linear regressions.

	Eggs					Larvae				
	Slope	F	t	R2	P	Slope	F	t	R2	P
**r_11_**	−0.0031	2	−1.4	0.13	0.18	0	0.16	−0.4	0.01	0.69
**r_22_**	−0.0019	0.6	−0.8	0.05	0.44	−0.002	1.4	−1.2	0.1	0.26
**r_33_**	−0.001	0.1	−0.3	0.01	0.75	−0.003	1.59	−1.3	0.11	0.23
**r**	−0.0015	0.3	−0.6	0.03	0.57	−0.003	1.6	−1.2	0.11	0.23

The dependent variables are the annual per-capita growth rates for pink bollworm density of one specific generation (*r_ii_*, i = 1,2,3) or for the average density of three generations (*r*). The independent variable is the percent Bt cotton (PBt).

### Within-year Pink Bollworm Growth Rates

**Figure 2:**
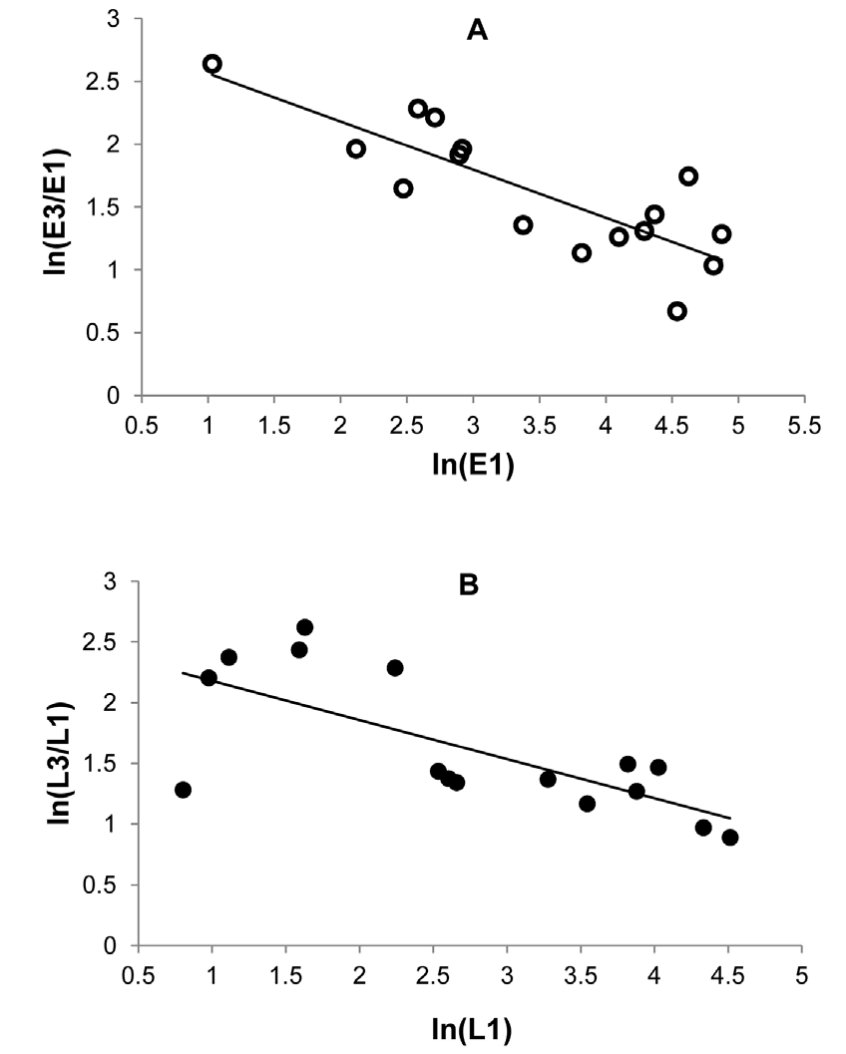
Within-year density dependence from G1 to G3. The per-capita pink bollworm growth rate from the first to the last generation within the same year, ln(E3/E1) or ln(L3/L1), declined significantly as the log of density increases, (**A**) for eggs (slope = −0.38, df = 14, R^2^ = 0.68, P = 0.0001) and (**B**) for larvae (slope = −0.32, df = 14, R^2^ = 0.51, P = 0.002).

Single-variable regression showed that the per-capita growth rate of pink bollworm from the first generation (G1) to the last generation (G3) within the same year, 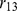, was negatively associated with the log density of G1, for eggs (Fig. 2A) and larvae (Fig. 2B). The linear regression is significant, for eggs (slope = −0.38, df = 14, R^2^ = 0.68, P = 0.0001) and larvae (slope = −0.32, df = 14, R^2^ = 0.51, P = 0.002). Meanwhile, the same per-capita growth rate 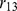 was positively associated with the percent Bt cotton, for eggs and larvae (Fig. S1 in File S1). The linear regression is significant, for eggs (slope = 0.010, df = 14, R^2^ = 0.59, P = 0.0005) and larvae (slope = 0.011, df = 14, R^2^ = 0.57, P = 0.0007).

Although 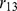 was significantly affected by both log density and percent Bt cotton, it might be better predicted by only one of these two variables. Stepwise regressions with one or both variables showed that this was the case. For both eggs and larvae, the per-capita growth rate 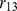 was better predicted by log density (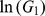) or percent Bt cotton (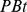) alone than by both variables together (determined by smallest RMSE, see Methods). Because there was no biological mechanism by which Bt cotton could directly result in increase in the within year growth rates, we considered log density as the only predictor variable for modeling. The regression equations for eggs (E) and larvae (L) are, respectively, as follows
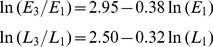
(1)


Regression equations with both log density and percent Bt cotton as the predictor variables were also derived (Appendix A in File S1). These equations were not used for modeling because 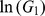 and 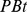 were highly correlated (correlation coefficient is −0.95 for eggs and −0.98 for larvae), which could result in erratic coefficients that cannot be properly explained (e.g. a positive coefficient for a predictor that had negative effects on the response variable).

**Figure 3:**
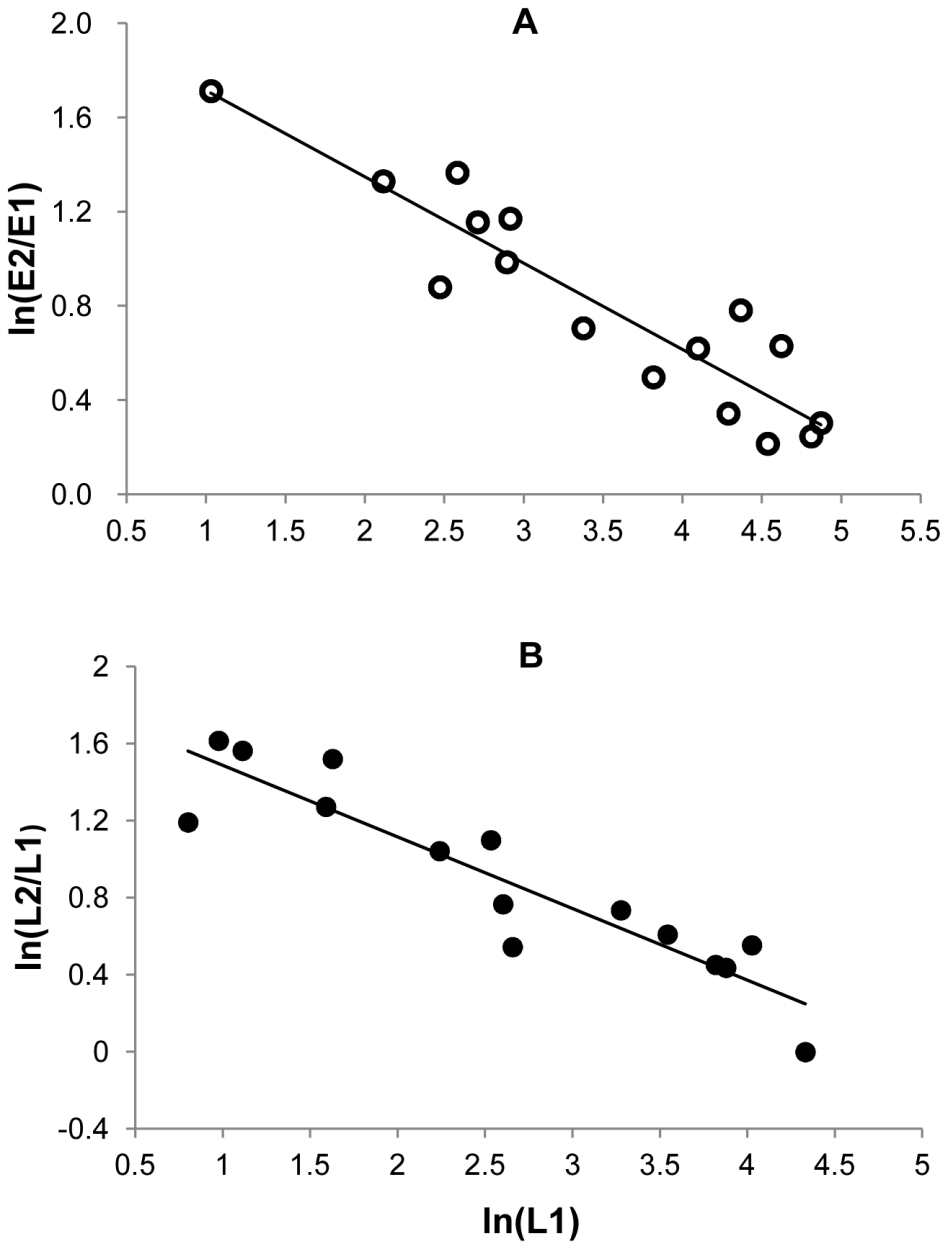
Within-year density dependence from G1 to G2. The per-capita pink bollworm growth rate from the first to second generation within the same year, ln(E2/E1) or ln(L2/L1), declined significantly as the log of density increases, (**A**) for eggs (slope = −0.37, df = 14, R^2^ = 0.85, P = 0.0001) and (**B**) for larvae (slope = −0.40, df = 14, R^2^ = 0.85, P = 0.0018).

Similar analyses were conducted for 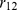, the within-year growth rate of pink bollworm from G1 generation to G2. Analyses showed that 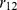 was negatively associated with the log density of G1, for eggs (Fig. 3A) and larvae (Fig. 3B). The linear regression is significant, for eggs (slope = −0.37, df = 14, R^2^ = 0.85, P<0.0001) and larvae (slope = −0.40, df = 14, R^2^ = 0.85, P = 0.002). As with the growth rate 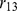, the growth rate 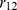 was positively associated with the percent Bt cotton (Fig. S2 in File S1). The linear regression is significant, for eggs (slope = 0.010, df = 14, R^2^ = 0.74, P<0.0001) and larvae (slope = 0.012, df = 14, R^2^ = 0.79, P<0.0001).

Stepwise regression analyses showed that for eggs and larvae, the within-year per-capita growth rate 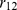 was better predicted by log density (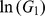) alone than by the percent Bt cotton (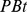) or both variables together (determined by smallest RMSE). The regression equations with log density as the only predictor variable were obtained as follows
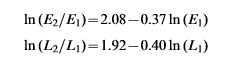
(2)


Unlike 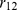 and 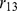, the within-year growth rate of pink bollworm from generation G2 generation to G3, 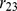, was not significantly associated with the log density in G2 generation, neither for eggs (slope = −0.0044, df = 14, R^2^ = 0, P = 0.95) nor for larvae (slope = 0.15, df = 14, R^2^ = 0.12, P = 0.19). The result suggests that the population growth of pink bollworm from generation G2 generation to G3 was not density-dependent.

### Cross-year Pink Bollworm Growth Rates

**Figure 4:**
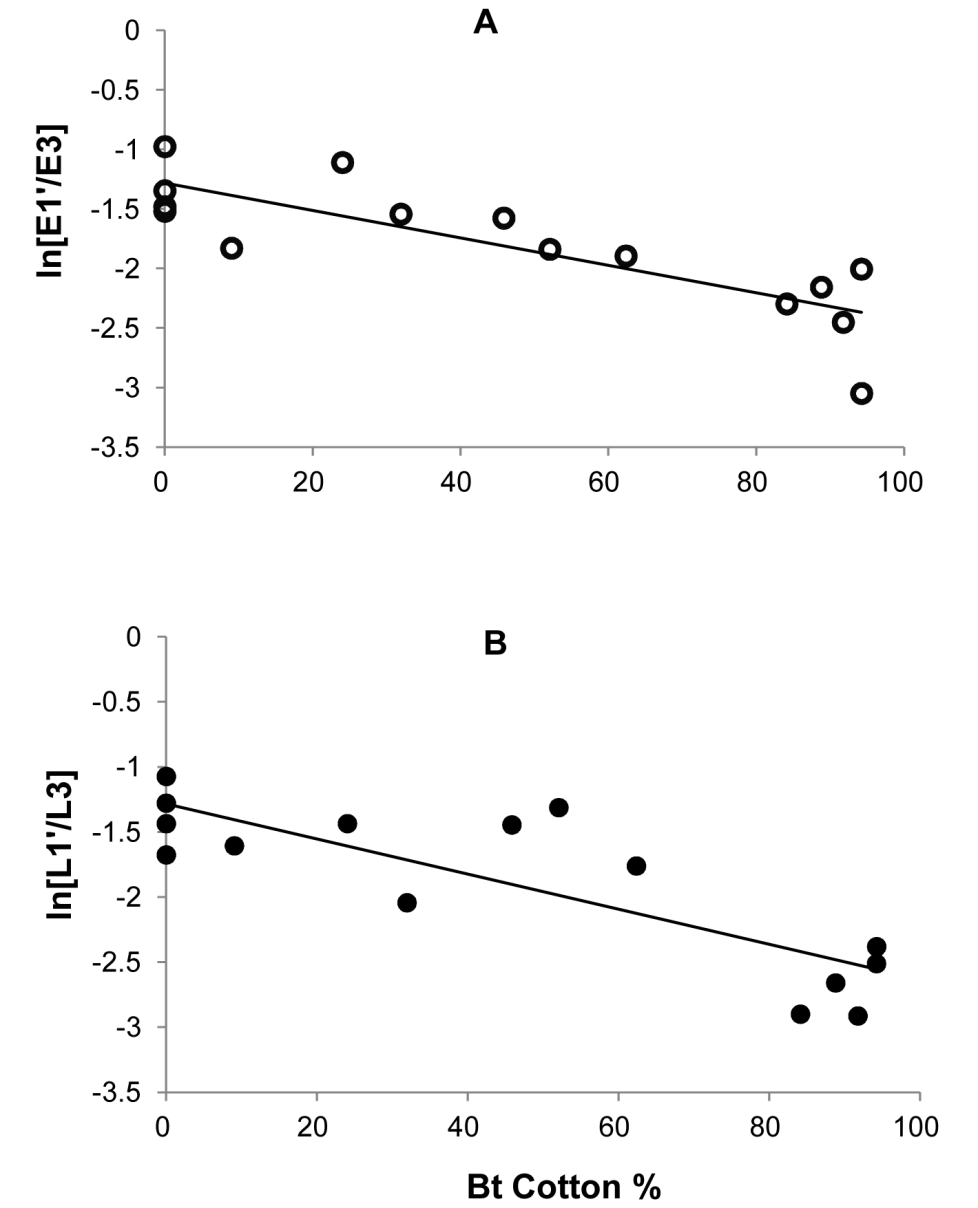
Cross-year growth rates of pink bollworm versus percent Bt cotton. (**A**) Eggs: Cross-year growth rate from last generation of the present year to the first generation of next year, ln(E1′/E3), versus Bt cotton %. Regression shows that ln(E1′/E3) decreases linearly as percentage of Bt cotton increases (slope = −0.012, df = 13, R^2^ = 0.68, P = 0.0002). (**B**) Larvae: Cross-year growth rate ln(L3/L1) versus Bt cotton %. Regression shows that ln(L3/L1) decreases linearly as percent Bt cotton increases (slope = −0.013, df = 13, R^2^ = 0.70, P = 0.0001).

The per-capita growth rate of pink bollworm from the last generation of the present year (G3) to the first generation of the next year (G1′), 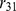, was negatively associated with the percent Bt cotton, for eggs (Fig. 4A) and larvae (Fig. 4B). The estimated slopes for eggs and larvae are −0.012 (df = 13, R^2^ = 0.68, P = 0.0002) and −0.013 (df = 13, R^2^ = 0.70, P = 0.0001), respectively. The per-capita growth rate 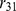 was positively associated with log density in the G3 generation. The linear regression is significant, for eggs (slope = 0.50, df = 13, R^2^ = 0.38, P = 0.012) and larvae (slope = 0.48, df = 13, R^2^ = 0.30, P = 0.033).

Stepwise regressions showed that for eggs and larvae, the cross-year per-capita growth rate 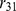 was better predicted by percent Bt cotton (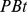) alone than by log density (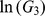) or both variables together (determined by the smallest RMSE). Therefore, we considered 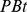 as the only predictor variable for modeling. The resulting regression equations were obtained as follows (where *E*′ and *L*′ represent population densities of eggs and larvae in the next year respectively):
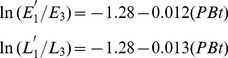
(3)


Regression equations with both log density and percent Bt cotton as the predictor variables were also derived (Appendix A in File S1). However, because 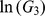 and 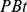 were highly correlated (−0.89 for eggs and −0.85 for larvae) the estimated coefficients were not reliable for prediction. Thus, these equations were not used for modeling.

### Equilibrium Pink Bollworm Population Density

While we have shown that the population density of pink bollworm declines over time as the percent Bt cotton increases, it is important to know what level of pink bollworm population density would remain when the percent Bt cotton was maintained at a specific constant. To this end we derived the equilibrium pink bollworm population density as a function of percent Bt cotton (Appendix B in File S1). Using the regression coefficients estimated in Eqs 1–3, the equilibrium egg and larval densities for G1, G2 and G3 generations were derived, respectively, as follows.
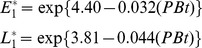
(4)

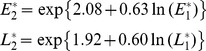
(5)

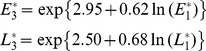
(6)


**Figure 5:**
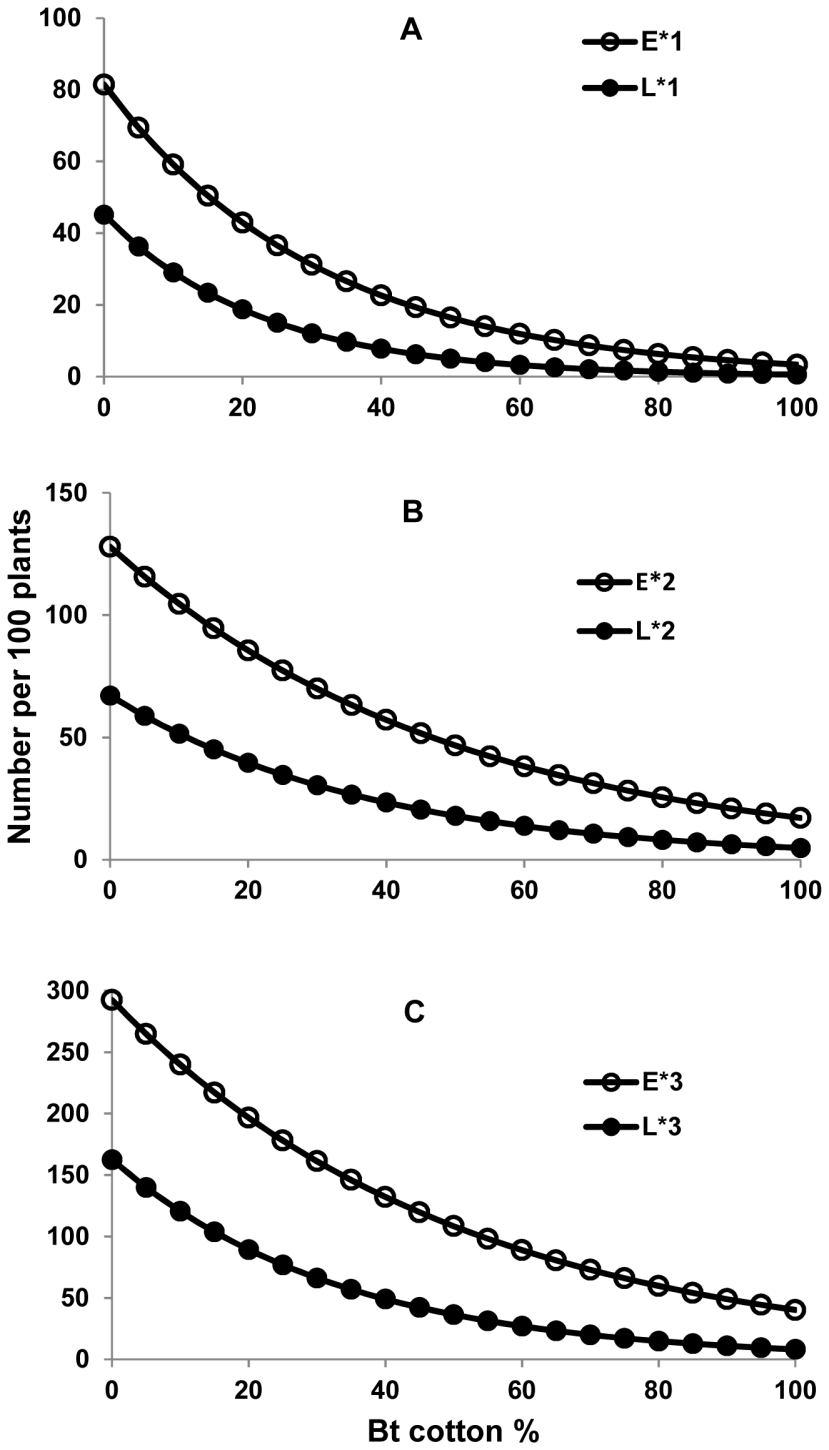
Model prediction: Equilibrium densities of pink bollworm eggs and larvae per generation as a function of percentage of Bt cotton. 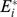
 and 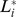 (i = 1,2,3) correspond to the egg and larval densities in the first (i = 1; panel (**A**)), second (i = 2; panel (**B**)) and third (i = 3; panel (**C**)) generations, respectively.

The equilibrium egg and larval densities in each of the three generations were predicted to decline exponentially as the percent Bt cotton increases (Fig. 5). Therefore, the rate of decline in equilibrium density became increasingly smaller as percent Bt cotton became higher. Simple calculations showed that compared to when there is no Bt cotton, 80% and 95% Bt cotton caused 82% and 87% reductions in the annual average egg density (i.e. 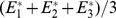), and 91% and 94% reduction in the annual average larval density (i.e. 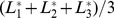), respectively. In other words, when the percent Bt cotton increased 15% from 80% to 95%, Bt cotton was predicted to cause only 5% more reduction in egg density and 3% more reduction in larval density.

## Discussion

Effectiveness of pest control by Bt crops can be affected by several factors such as the proportion of Bt crops to non-Bt crops, the net reproductive ratio of pest on non-Bt cotton, the efficacy of Bt cotton and pest migration rate between Bt plants and non-Bt plants [13]. While each of these factors has been examined carefully in the literature, less attention has focused on density dependence that may potentially enhance or diminish the impact of Bt crops. By analyzing 16 years of field data of pink bollworm abundance per generation and percentage of Bt cotton in the Yangtze River Valley of China we showed that the net effects of Bt cotton on suppression of pink bollworm became more limited as percent Bt cotton became increasingly higher. Our analyses that separated within-year dynamics from cross-year dynamics showed that the diminished effects of Bt cotton on suppression of pink bollworm were associated with a direct, within-season density dependence (*i.e.* increased growth rates within the cotton growing season when pest density was lower).

Reduced insecticide sprays might be one of the potential factors leading to higher growth rate within the season when pest densities became lower. Because Bt cotton varieties in China are not sufficiently efficacious to kill all susceptible insects, insecticide sprays were used by farmers to augment pest suppression [3]. Insecticide sprays were gradually reduced as the percentage of Bt cotton increased in the Yangtze River Valley. The average insecticide sprays targeting bollworms declined from an average of 8.0 per season during the pre-Bt period (1992–1999) to 2.5 in 2010 [22]. The lower number of insecticide sprays could generally favor pest growth. However, insecticide sprays did not change substantially during 2006–2010 when the percentage of Bt cotton was greater than 84%. The number of insecticide sprays declined from 3.4 in 2006 to 2.5 in 2010, less than 0.25 per year [22]. Therefore, insecticide sprays might have played only a limited role as a density dependent factor, at least when the percentage of Bt cotton was high.

Natural enemies are another potential factor that could contribute to direct density dependence within the season. In the Yangtze River Valley, pink bollworm is attacked by a number of parasitoid wasps during the cotton growing season. Some of them (e.g. *Bracon nigrorufum* (Cushman) and *Bracon isomera* (Cushman)) almost only parasitize pink bollworm. The general dynamics of host-parasite interactions suggest that when the density of pink bollworm was reduced by Bt cotton, the density of the specialized parasitoid wasps would decline more precipitously. Therefore, pink bollworm populations with a lower density would be favored to grow because of a lower rate of parasitism. Whatever the reason, the direct density dependence for pink bollworm within the season appears to have been manifested in the Yangtze River Valley.

The diminishing effects of Bt crops on pest control may be a more general ecological phenomenon in Bt crop-based pest suppression. Wu et al. (2008) have shown that field densities of cotton bollworm (*Helicoverpa armigera*) in Northern China were significantly reduced since Bt cotton was commercially planted across the region in 1997. The Bt cotton planting area in the region had gradually increased over the years. By carefully examining their 10-year data from 1997 to 2006 [16] one finds that the egg and larval densities of cotton bollworm almost remained constant in the later four years from 2003 to 2006. This trend is similar to what we observed here for pink bollworm.

In another system in the United States, field densities of European corn borer (*Ostrinia nubilalis*) in three states, Minnesota, Illinois and Wisconsin, have been observed to decline significantly since Bt maize was planted in 1997 [17]. The time series data, especially those in Minnesota clearly showed that the pest densities were relatively constant during 2004–2010 when Bt maize was intensively planted [17]. A separate analysis suggested that *Nosema pyrausta*, an entomopathogenic parasite to *O. nubilalis*, was responsible for the cyclic dynamics in pest density while Bt maize served as “brakes” for reducing the amplitude of cycles [23]. The two additional examples described above suggest that the diminishing effects of Bt crop on pest control revealed here is not a specific case.

The benefits of Bt cotton in China reported here and in [22] for suppressing pink bollworm parallel previously reported benefits of Bt cotton in China for suppressing cotton bollworm on non-Bt host plants [16]. However, early evidence of resistance to the Cry1Ac toxin produced by Bt cotton grown in China has been reported for both pests [24], [25]. Options for reducing the negative consequences of this resistance in China include switching to a different Bt toxin, using Bt cotton that produces two or more toxins, and increasing the percentage of non-Bt cotton [24]–[26]. Deciding the best strategy requires an overall assessment of both pest suppression efficiency and pest resistance risk. Economic thresholds also need to be taken into account. The quantitative results presented here are an important part of the assessment that will help determine an optimal strategy ultimately. Our study only examined patterns of abundance. Future manipulative experimental studies are needed that investigate the causes of the negative relationship between pest density and population growth in Bt crop systems.

## Materials and Methods

### Percentage of Cotton Planted with Bt Cotton

The hectares planted to Bt cotton in the six provinces of the Yangtze River Valley were obtained from the Chinese Agricultural Ministry (2000–2010) and the total hectares of cotton planted were from the China Agriculture Yearbook. The percentage of Bt cotton for each year was calculated as the hectares of Bt cotton divided by the total hectares of cotton times 100. More details about the varieties of Bt and conventional cotton were reported previously [22], [24].

### Abundance of Pink Bollworm Eggs and Larvae Per Generation

During 1995–2010, the abundance of pink bollworm eggs and larvae on cotton was monitored in six provinces of the Yangtze River Valley as part of routine national monitoring [22]. Larvae were collected from plants in Non-Bt fields while eggs were collected from Bt and Non-Bt fields. Because pink bollworm adults do not discriminate between Bt and Non-Bt plants when selecting sites for oviposition, the data on these two stages provide complimentary information. Egg densities in each generation are a snapshot of the areawide abundance while larval densities reflect more of the local pest abundance in Non-Bt fields. In this region, pink bollworm has three generations per year [27]. Usually, the first generation appears in July when cotton plants are flowering, the second generation occurs from early August to middle September when cotton bolls appear, and the third generation occurs in late September through October.

Within the cotton growing season of each year from 1995 to 2010, the abundance of pink bollworm eggs and larvae on non-cotton plants was sampled from variable sites across the region. The abundance of pink bollworm was measured in different ways for the three generations but all measurements were transformed to the number of pink bollworms per 100 plants. Detailed methods of sampling and measurement were reported previously [22], [28].

For each generation in each year, we calculated the provincial mean population density based on densities at all non-Bt sampling sites in the province. The annual regional population density for the Yangtze River Valley for each generation was calculated as the mean of the annual provincial population densities for that generation in that year.

### Within-year, Cross-year and Annual Per-capita Growth Rates

Per-capita growth rate is an important quantity measuring population growth potential [29]. Here we defined three types of per-capita growth rate for pink bollworm. First, we defined the per-capita growth rate for pink bollworm from generation 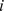 to 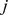 (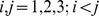) within the same year as 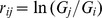, where 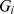 and 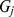 represent the population densities of generation 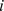 and 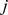 within the same year, respectively. Among these growth rates, our primary concern is 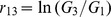, which measures pink bollworm growth potential during the entire cotton growing season.

Second, we defined the cross-year per-capita growth rate for pink bollworm from the last generation of present year (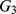) to the first generation of next year (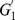) as 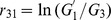.

Finally, we defined the annual per-capita growth rate for generation 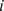 as 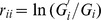 (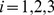). Our primary concern is 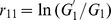, which measures the per-capita growth rate for G1 from one year to the next. Because 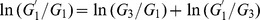 we find that 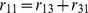, which means that the annual per-capita growth rate for G1 can be additively decomposed into the within-year growth rate from G1 to G3 within the same year and cross-year growth rate from G3 in one year to G1 in the next year. Note that the annual per-capita growth rate can be defined for the average density of pink bollworm across all three generations, which is commonly denoted as 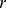.

### Model Analysis

One way to predict the stable population density of pink bollworm for a given percent Bt cotton is directly establishing the statistical relationship between the annual per-capita growth rate and percentage of Bt cotton. However, this cannot be achieved because the regression of annual growth rate against percent Bt is not significant (see Table 1). Alternatively, we decompose the annual growth rate into a within-year and a cross-year growth rate, and derive a population dynamic model of pink bollworm that combines the within-year dynamics with the cross-year dynamics. The model then is used to predict the stable equilibrium density of pink bollworm for any given percent of Bt cotton. The detailed derivation of the model is given in Supporting Information (File S1).

### Stepwise Regression

We used stepwise regression (*stepwise* in MATLAB (2010a)) to determine the appropriate regressive model for the within-year growth rates and cross-year growth rates. This approach determines the best regression model by stepwise addition of the most significant explanatory variables and removal of non-significant explanatory variables [30]. The final (best) model is determined by the smallest RMSE (root mean square error).

## Supporting Information

File S1 **A combined file containing Appendix A & B; Fig. S1 & S2.**
(PDF)
