# Supplementary material for: Diminishing Returns from Increased Percent Bt Cotton: The Case of Pink Bollworm
Source: PLoS One. 2013 Jul 16;8(7):e68573. doi: 10.1371/journal.pone.0068573 (PMC3713026; doi:10.1371/journal.pone.0068573)
Supplement: File S1 — A combined file containing Appendix A & B; Fig. S1 & S2. (PDF) [file pone.0068573.s001.pdf]

Supporting Information

Diminishing returns from increased percent Bt Cotton: The case of pink bollworm

Yunxin Huang[1], Peng Wan[2], Huannan Zhang[1], Minsong Huang[2], Zhaohua Li[1], Fred Gould[3]

[1]School of Resource and Environmental Science, Hubei University, Wuhan, P. R. China,

[2]Institute of Plant Protection and Soil Science, Hubei Academy of Agricultural Sciences, Wuhan, P. R. China,

[3]Department of Entomology, NC State University, Raleigh, North Carolina, United States of America

Appendix A: Multiple regression analyses

This supplemental section presents the results of multiple regressions with both density and percent Bt cotton as the predictor variables.

Multiple regressions for the within-year growth rate r_{13} with percent Bt cotton (PBt) and log density ($\ln(E_1)$ or $\ln(L_1)$) as the predictor variables are significant (R^2=0.69, P=0.0005 for eggs and R^2=0.59, P=0.003 for larvae). The resulting regression equations are obtained as follows

$$\begin{aligned} \ln(E_3/E_1) &= 3.30 - 0.46\ln(E_1) - 0.0023(PBt) \\ \ln(L_3/L_1) &= 0.087 + 0.27\ln(L_1) + 0.019(PBt) \end{aligned} \qquad \text{A.1}$$

Because the two predictor variables are highly correlated (correlation coefficient of -0.95 for eggs and -0.98 for larvae), multiple regressions here involve collinearity which results in erratic coefficients like the positive sign for log density in the second equation of (A.1). Thus, the regression equations in (A.1) are not used for modeling.

Multiple regressions for the within-year growth rate r_{12} with two predictor variables, the percent Bt cotton (PBt) and log density, are significant (R^2=0.85,

P=0.0001 for eggs and R^2=0.86, P=0.0001 for larvae):

$$\begin{aligned}\ln(E_2/E_1) &= 2.29 - 0.41\ln(E_1) - 0.0013(PBt)\\ \ln(L_2/L_1) &= 2.58 - 0.56\ln(L_1) - 0.0052(PBt)\end{aligned} \quad \text{A.2}$$

Multiple regressions for the cross-year growth rate r_{31}, with both percent Bt cotton (*PBt*) and log density ($\ln(E_3)$ or $\ln(L_3)$) as the predictor variables, yield the following equations

$$\begin{aligned}\ln(E_1^{'}/E_3) &= 0.51 - 0.016(PBt) - 0.30\ln(E_3)\\ \ln(L_1^{'}/L_3) &= 2.93 - 0.027(PBt) - 0.80\ln(L_3)\end{aligned} \quad \text{A.3}$$

Where *E'* and *L'* represent population densities of eggs and larvae in the next year, respectively. Regressions are significant, for eggs (R^2=0.71, P=0.0005) and larvae (R^2=0.88, P=0.0001).

Appendix B: the population dynamics of pink bollworm

This supplemental section presents the detailed derivation of the model used for predicting the long-term equilibrium density of pink bollworm for a given percent of Bt cotton. The model combines the within-season dynamics with the overwintering dynamics of pink bollworm. The steps of derivation are as follows.

First, we assume that the within-year growth rate from generation G1 to G3 depends linearly on pest density ($\ln(G_1)$) and percentage of Bt cotton (PBt):

$$\ln(G_3/G_1) = a_{13} + b_{13}\ln(G_1) + c_{13}(PBt) \quad \text{B.1}$$

Next, we assume that the cross-year growth rate from generation G3 to G 1 depends linearly on pest density ($\ln(G_3)$) and percentage of Bt cotton (PBt)

$$\ln(G_1^{'}/G_3) = a_{31} + b_{31}\ln(G_3) + c_{31}(PBt) \quad \text{B.2}$$

Summing up (B.1) and (B.2), we find that

$$\ln(G_1^{'}/G_1) = (a_{13} + a_{31}) + b_{13}\ln(G_1) + b_{31}\ln(G_3) + (c_{13} + c_{31})(PBt) \qquad \text{B.3}$$

(B.3) can be rewritten as

$$G_1^{'} = G_1 \exp\{(a_{13} + a_{31}) + b_{13}\ln(G_1) + b_{31}\ln(G_3) + (c_{13} + c_{31})(PBt)\} \qquad \text{B.4}$$

Where $\ln(G_3) = a_{13} + (1 + b_{13})\ln(G_1) + c_{13}(PBt)$.

For a given percentage of Bt cotton (PBt), (B.4) is a recursive equation for G_1.When $G_1^{'} = G_1 = G_1^{*}$, (B.4) has an equilibrium at

$$G_1^{*} = \exp\left\{-\frac{[a_{13}(1 + b_{31}) + a_{31}] + [c_{13}(1 + b_{31}) + c_{31}](PBt)}{b_{13}(1 + b_{31}) + b_{31}}\right\} \qquad \text{B.5}$$

Which predicts the stable equilibrium density for a given percent of Bt cotton. The parameters $a_{13}, b_{13}, c_{13}, a_{31}, b_{31}, c_{31}$ need to be estimated by linear regressions.

Once the equilibrium for G1 is determined, the equilibrium for G3 can be derived from (B.1) as

$$G_3^{*} = \exp\{a_{13} + (1 + b_{13})\ln(G_1^{*}) + c_{13}(PBt)\} \qquad \text{B.6}$$

Finally, we assume that the within-year growth rate from G1 to G2 depends linearly on pest density ($\ln(G_1)$) and percentage of Bt cotton (PBt):

$$\ln(G_2/G_1) = a_{12} + b_{12}\ln(G_1) + c_{12}(PBt)$$

In which the equilibrium for G2 is expressed as a function of G_1^{*} and PBt as follows

$$G_2^{*} = \exp\{a_{12} + (1 + b_{12})\ln(G_1^{*}) + c_{12}(PBt)\} \qquad \text{B.7}$$

The annual average equilibrium is $G^{*} = (G_1^{*} + G_2^{*} + G_3^{*})/3$, which is a function of PBt.

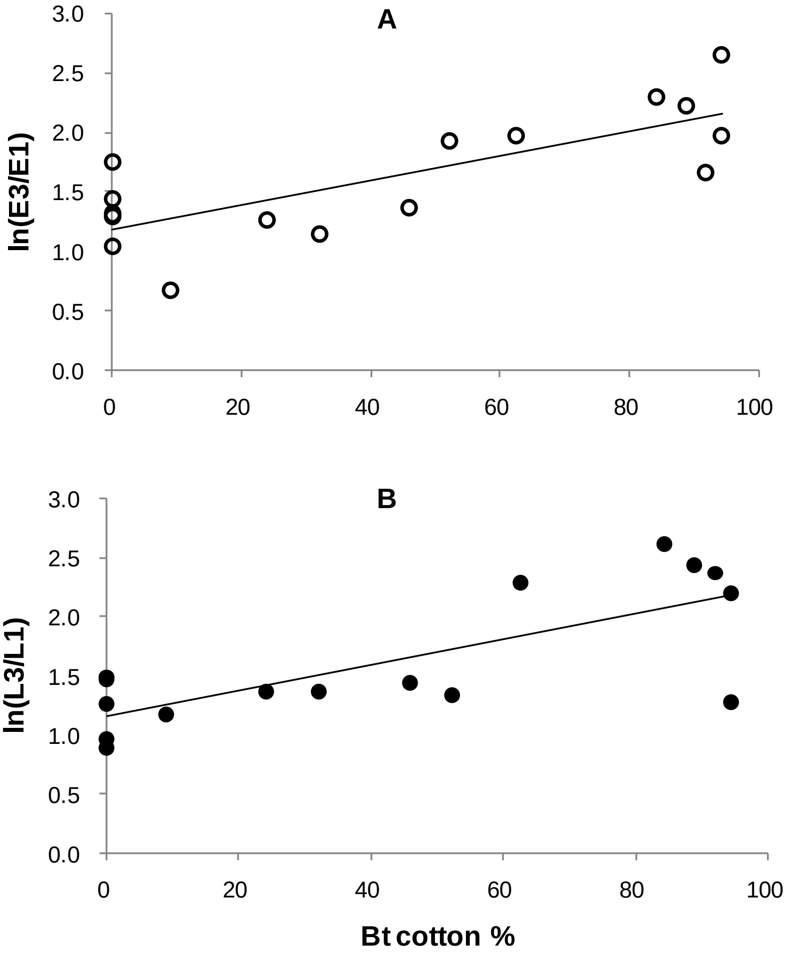

Fig. S1. Per-capita growth rates of pink bollworm from G1 generation to G3 within the same year versus percent Bt cotton, for eggs (A) and larvae (B). The linear regression is significant, for eggs (slope=0.010, df=14, R^2=0.59, P=0.0005) or larvae (slope=0.011, df=14, R^2=0.57, P=0.0007).

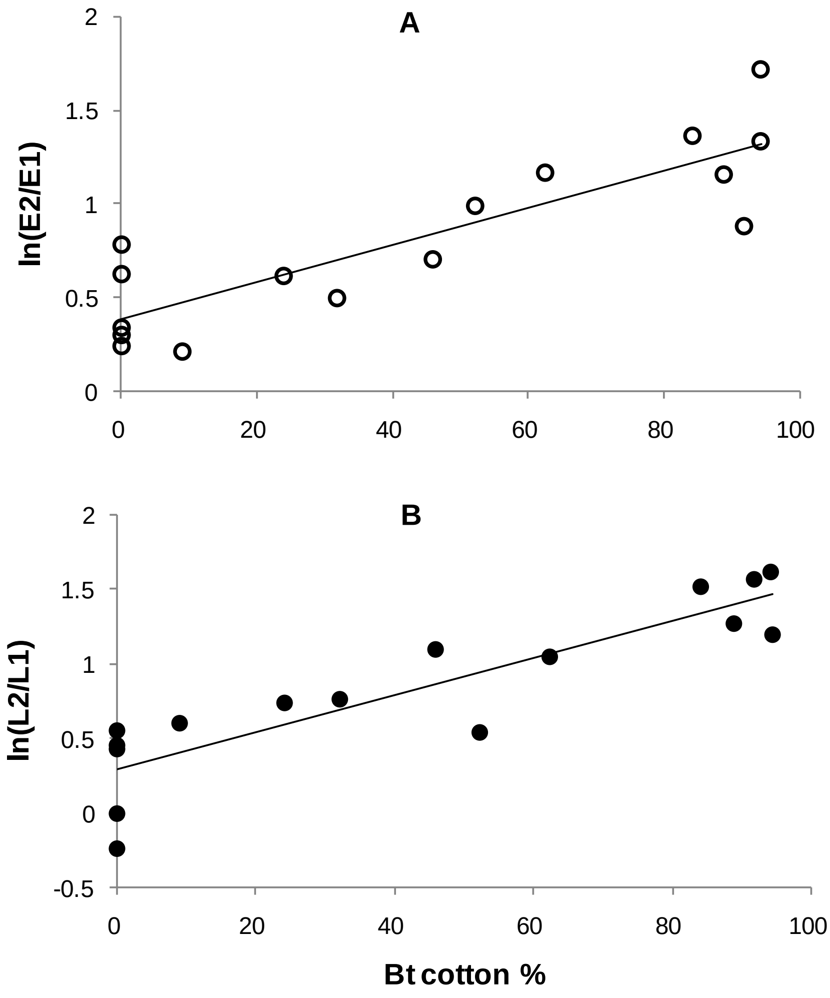

Fig. S2. Per-capita growth rate of pink bollworm from G1 generation to G2 within the same year versus percent Bt cotton, for eggs (A) and larvae (B). The linear regression is significant, for eggs (slope=0.010, df=14, R^2=0.74, P<0.0001) and larvae (slope=0.012, df=14, R^2=0.79, P<0.0001).
